# Body awareness in persons diagnosed with rheumatoid arthritis

**DOI:** 10.3402/qhw.v9.24670

**Published:** 2014-10-31

**Authors:** Helena Lööf, Unn-Britt Johansson, Elisabet W. Henriksson, Staffan Lindblad, Jennifer Bullington

**Affiliations:** 1 Sophiahemmet University, Stockholm, Sweden; 2 Department of Clinical Science and Education, Södersjukhuset, Karolinska Institutet, Stockholm, Sweden; 3 Department of Neurobiology, Care Sciences and Society, Division of Nursing, Karolinska Institutet, Stockholm, Sweden; 4 Department of Rheumatology, Karolinska University Hospital, Stockholm, Sweden; 5 Department of Learning Informatics, Management and Ethics, Karolinska Institutet, Stockholm, Sweden; 6 Department of Health Care Sciences, Ersta University College, Stockholm, Sweden

**Keywords:** Body awareness, phenomenology, life-world perspective, lived experience, rheumatoid arthritis

## Abstract

Living with rheumatoid arthritis (RA) poses physiological and psychological demands on a person. RA is a autoimmune disease that can cause pain, disability, and suffering. The ability to notice bodily inner sensations and stimuli (body awareness, BA) is described in the literature in ways that could have either a positive or a negative impact on a person’s health. The concept of BA is complex and a thorough understanding is needed about what BA means from the patient’s perspective. This study was therefore conducted to acquire greater insight into this phenomenon. The study is grounded in a phenomenological life-world perspective. Eighteen narrative interviews were conducted in patients (age range 23–78 years) with RA. The interviews were analyzed using the Empirical Phenomenological Psychological method. General characteristics were found running through all 18 interviews, indicating that the disease resulted in a higher degree of negatively toned BA. BA was either a reactive process of searching or controlling after disease-related symptoms or a reactive process triggered by emotions. BA was an active process of taking an inventory of abilities. All participants had the ability to shift focus from BA to the outside world. Four typologies were identified: “A reactive process on symptoms,” “A reactive process on emotional triggers,” “An active process of taking an inventory of abilities,” and “A shifting from BA to the outside world.” In conclusion, because BA can be both positively and negatively toned, health care professionals must have a good understanding of when BA is positive and when it is negative in relation to the patient. RA had caused a higher degree of negatively toned BA. Thus, the ability to shift attention from BA to activity in the outside world could sometimes be beneficial for the patient’s general health.

Rheumatoid arthritis (RA) is a systemic autoimmune disease that results in a chronic, systemic inflammatory disorder that typically occurs in the small joints of the hands and feet. RA subsequently spreads to the larger joints, causing joint deformity and progressive physical disability (Symmons, 2002). People with RA have an increased risk of premature death, due to cardiovascular disease, possibly related to inflammation (Akrout et al., 2012) and other factors (Pereira & Borba, 2008). A classic symptom of RA is morning stiffness that usually lasts for more than an hour (Symmons, 2002). Fatigue and pain are two major concerns of patients (Carr et al., 2003; Heiberg, Finset, Uhlig, & Kvien, 2005). Fatigue is expressed as an enigma that is difficult to describe and explain, but has been depicted as a disturbing and serious problem that often prevents a person from participating in enjoyable and meaningful activities (Rasker, 2009). Furthermore, depression is highly prevalent in RA and is associated with poorer RA outcomes (Matcham, Rayner, Steer, & Hotopf, 2013). Disease symptoms of RA can affect an individual’s daily life (Ahlstrand, Björk, Thyberg, Börsbo, & Falkmer, 2012). Treatment goals strive to reduce mortality, relieve/reduce pain, and prevent loss of function (The National Board of Health and Welfare, 2012). Findings indicate the need to recommend and support health-enhancing physical activity behaviors in people with RA (Eurenius & Stenström, 2005; Stenström & Minor, 2003). Melanson and Downe-Wamboldt (2003) and Tak (2006) found that physical limitations in daily life provide the largest disease-related worries for the patients. RA has been shown to influence a person’s mood and social life; in particular, feelings of helplessness and uncertainty can lead to anger and depression (Lutze & Archenholtz, 2007). Mental requirements and an overall need for coping management strategies are important (Engelbrecht, Wendler, & Alten, 2012; Gronning, Lomundal, & Koksvik, 2011; Shariff et al., 2009). Shariff et al. (2009) demonstrated that for those living with pain, a sense of well-being is achieved not only through pain control alone but also through various mind/body techniques for managing pain, accepting new limits, and adjusting the way people relate to themselves (Shariff et al., 2009). Finding the right activity balance could be a struggle because it is easy to be overactive, which would trigger elevations in pain and reduced vigor. Participants described activities as a mediator of pain and a distraction from it (Ahlstrand et al., 2012). McDonald et al. (2012) observed that the participants in their study experienced uncertainties associated with periods of disease flare-ups, but that they also had good days, bad days, and worse days. Kowalski and Chung (2012) suggest that coping strategies should not be meticulously planned in advance, but rather people should think positively and participate in activities regarded as enjoyable or amusing (Kowalski & Chung, 2012).

The tendency to focus attention on bodily internal sensations and stimuli is referred to as body awareness (BA) (Ingram, 1990). BA, which includes cognitive, emotional, and perceptual abilities, is the bodily aspect of a person’s overall self-awareness (Roxendal & Winberg, 2002). BA is a complex phenomenon, having either a positive or a negative impact on a person’s health and well-being (Mehling et al., 2009). BA may lead to an obsession or excessive dwelling on bodily functions (Abramowitz, Schwartz, & Whiteside, 2002; Kirmayer & Looper, 2006). Studies have demonstrated that BA is strongly associated with unfavorable clinical outcomes, such as the trajectory of long-term, chronic low back pain. Psychological factors, notably distress, depressive mood, and somatization, have been implicated in the transition to chronic low back pain (Pincus, Burton, Vogel, & Field, 2002). Investigations have also exposed the advantages regarding the ability to notice subtle body sensations and that it may be useful in the management of chronic diseases, such as congestive heart failure (Baas, Berry, Allen, Wizer, & Wagoner, 2004), chronic renal failure (Christensen, Wiebe, Edwards, Micheles, & Lawton, 1996), and chronic low back pain (Mehling, Hamel, Acree, Byl, & Hecht, 2005). Improved BA may change the attitude to one’s own body, enhance the ability to listen to bodily symptoms, and increase sense of control in daily life. Moreover, improved BA may provide an understanding in the ability to manage chronic pain (Gard, 2005; Gustafsson, Ekholm, & Broman, 2002; Gyllensten & Gard, 2008). Understanding of BA has been applied to studies of pain by using distraction techniques. Attentional focus is then directed away from the pain sensations and towards mental tasks. By using a pain-inducing activity (e.g., solving mental tasks), certain benefits would be expected (e.g., reduced pain perception while attention is distracted away from noxious stimuli) (Banestick et al., 2002; Johnson, 2005). Some ancillary evidence suggests that distraction should be used with caution; for instance, findings show that distraction from chronic pain (during a pain-inducing activity) is associated with greater post-activity pain (Goubert, Crombez, Eccleston, & Devulder, 2004). Theoretically, an increase in BA may serve to help individuals recognize symptoms of worsening RA. However, it is not known whether increased BA in persons with RA leads to somatization, an abnormal dwelling on body symptoms. Because many symptoms of RA (e.g., pain and fatigue) are generally evocative of negative emotional responses, elevated BA may be associated with poor emotion regulation and worse perceived general health. Lööf, Johansson, Welin Henriksson, Lindblad, and Saboonchi (2013) reported that severe fatigue in RA was associated with increased BA and decreased positive affect. The concept of BA, as well as self-reported measures within the concept, is used for a wide range of diseases. Mehling et al. (2009) stressed the need for a clear definition and an in-depth understanding of the concept of BA. Although a number of phenomenological studies have been carried out on persons with RA, these studies have not focused specifically on BA. The most common issues investigated are lived experiences of RA (Iaquinta & Larabee, 2004; Ryan, 1996), fatigue (Ream & Richardsson, 1997; Söderberg, Lundman, & Norberg, 2002), and specific populations such as mothers (Mitton, Treharne, Hale, Williams, & Kitas, 2007) or Korean women (Jeong Hwang, Hae Kim, & Sook Jun, 2004). The science of phenomenology has the advantage of encompassing the entire life-world of the person, including their bodily experiences, emotions, cognitions, and social experiences. The work of Merleau Ponty (1945/1962) on the “lived body” as the mind-body-world nexus provides an excellent inspiration for understanding BA. The way in which persons perceive their bodies, understand their life situations, perceive the world around them, and relate to other people is intricately interwoven in their experiences. Because no studies have focused specifically on BA in persons with RA, there is an urgent need to explore this phenomenon. This is one reason why a phenomenological study on the experience of BA in persons with RA can complement purely medical and psychological perspectives with a holistic understanding.

## Methods

The study was grounded in a phenomenological life-world perspective. This perspective includes the world in which we live and to which we ascribe meaning. The way a person perceives the world is always subjective and relative. In a phenomenological life-world perspective the focus is on the human experience (Spiegelberg, 1982). Karlsson’s (1993) Empirical Phenomenological Psychological (EPP) method was used in this study. Phenomenology, which is rooted in the philosophical tradition, was developed by Husserl (1962) to explore and find understanding for people’s experiences of their life-world. In empirical studies this means that the researcher aims to determine the essence and constituents of various human phenomena (Karlsson, 1993). Here, the meaning of people’s lived experiences in relation to the phenomenon of BA was examined through individual interviews in which the starting point was a genuine open attitude towards another person’s experiences and life-world. Each individual perceives and interprets the world in a unique way. A semi-structured interview guide was applied to stimulate narratives within the frame of the study’s focus. No leading questions were used, but rather open-ended questions that focused on experiences relevant to the aim of the study. According to the EPP method (Karlsson, 1993), the researcher does not attempt to validate hypotheses, nor does he or she attempt to prove theoretical constructions. Rather, importance is invested on openness to that which shows itself through the participants’ experiences. Consequently, to be open to the phenomenon as presented, the researcher must put aside all presumptions and theories about the phenomenon under investigation (Karlsson, 1993). To penetrate the phenomenon of BA the participants were asked the following questions:

Can you tell me about when you are aware of your own body? How do you pay attention to your body? What parts of your body do you recognize? Can you remember a situation when you noticed that your body felt comfortable and without problems? Can you remember a situation when you noticed that your body felt uncomfortable and disturbing? In what situations do you not pay attention to your body, and in what situations will body awareness disappear altogether? What does it mean for you to pay attention to your body?

### Participants

Rheumatologists at two university hospitals in Stockholm, Sweden, selected the participants. The rheumatologists chose the participants based on their ability to communicate valuable information about BA from their own perspective. Eligible for inclusion were patients aged 20–100 years with a confirmed diagnosis of RA according to the American College of Rheumatology classification criteria (Arnett et al., 1988). Twenty patients who formed a balanced heterogeneous group and fulfilled the inclusion criteria were selected. An information letter was initially given to the potential participants by the rheumatologists at the two university hospitals; in some cases (and in agreement with the potential participants) the letter was posted. A few days later after the potential participants had been given information about the study, the first author (HL) telephoned and offered additional information and then asked potential participants for their consent to participate. Two persons (both men) declined to participate (one cancelled the interview because of emotional stress and the other failed to show up for the prearranged interview). This person later phoned to say he had forgotten the appointment. The final sample included 18 patients (2 males and 16 females aged 23–78 years) with a diagnosed RA (disease duration 6–34 years). Two participants had basic education, eight had college education and eight had university education. Seven patients were living alone and 11 cohabiting. Patients with language difficulties or other problems that could interfere with participating in an interview were excluded.

### Interviews and settings

Eighteen narrative interviews, all performed from June to October 2013, were conducted. Audiotaped interviews took place according to the participants’ wishes at the following places: four interviews were conducted in the participants’ private office, one in HL’s private office, and 13 at the rheumatology clinics. The interviews took place in a private and quiet room, and the interviews lasting on average about 75 min. All 18 audio taped interviews were transcribed verbatim by HL. The verbatim transcription occurred within 2–5 days from the time the interviews were conducted.

### Data analysis

The analysis was performed using the EPP method (Karlsson, 1993) to study the phenomenon of BA. The research group met several times worked and discussed the data, arguing for the interpretations. E-mails with tracked text were sent back and forth within the research group between meetings. Data analysis was based on the following steps:

Step 1: A reading to gain an initial understanding of the text at a level of everyday understanding. HL read the entire transcribed interviews and separately listened to the tape recordings several times for each of the 18 participants.

Step 2: Interview texts were divided into meaning units, marking (directly in the text) where a change in meaning occurred in the text.

Step 3: Meaning units were transformed into the researcher’s language with focus on the significance of the meaning in relation to BA.

Step 4: The transformed meaningful units were assembled as an individual structure (situated structure) in terms of a synopsis, one structure for each individual interview.

Step 5: The condensed structures of each interview were synthesized into a general characteristic common to all (18) narrative accounts. Next, the variations of the phenomenon were interpreted in terms of “typological structures.” Each typology was exemplified by the participants’ quotations. The general characteristics were those constituents that were common throughout all the narratives, whereas the variations described the different ways in which the phenomenon appeared in the data. The aim was not to find “essences” but instead to maintain the empirical in its variation in an effort to gain a nuanced understanding of experience. As in all qualitative studies, our pre-understandings were purposely contained in order to allow possible new meanings to emerge from the texts. HL assumed the role of a researcher and had no direct patient contact as a nurse.

## Ethical consideration

The study was performed in accordance with the ethical principles of the Declaration of Helsinki (World Medical Association Declaration of Helsinki, 2013). Ethical approval was obtained from the ethics committee of the Regional Ethical Review Board (Dnr. 2013/718-31/2). All participants gave written informed consent and were informed of their right to discontinue their participation at any time. The participants’ anonymity and confidentiality were maintained throughout the study.

## Findings

### General characteristics

No general structure was found, which was expected due to all the various meanings contained in the 18 interviews. However, some general characteristics were present in the 18 interviews. The process of BA can be understood in terms of a searching for or the controlling of different symptoms. All 18 participants described BA as a reactive process emanating from their pain and other disease symptoms. The disease symptoms triggered negatively toned BA. Heightened BA was also described as a reactive process resulting from different emotional triggers. For some participants, heightened BA was connected to loss of control, feelings of anger, sadness and fear, worries about symptom aggravation, sense of grief over their weak and sick body, or feelings of being a burden to others. Other emotional triggers included participants connecting to physical bodily strength, accepting the disease, feeling a sense of community with others, and a feeling of being cared for or tranquility in body signals. These emotional triggers gave either heightened positively toned BA or negatively toned BA. Furthermore, BA was an active process initiated through the participants taking an inventory of their abilities. This heightened BA was performed as a means to examine the bodily abilities in relation to different planned activities. Finally, another general characteristic was that all participants had the ability to change focus from BA to the outside world. This change in focus seemed to occur when the participants experienced an enjoyable, interesting, or meaningful environment. There were, however, differences in the interview descriptions and therefore we have chosen to present the following four typologies: “A reactive process on symptoms,” “A reactive process on emotional triggers,” “An active process of taking an inventory of abilities,” and “A shifting from BA to the outside world.” Each typology is exemplified by the participants’ own descriptions.

## Typology I

### A reactive process on symptoms

In this typology emphasis is on listening to the different bodily signals in an effort to explore changes in disease-specific symptoms. The participants described that living with a disease and disease-related symptoms had been responsible for a negatively toned BA. The symptoms of the disease called for the person’s attention. Some participants noted that the symptoms had increased or decreased while others questioned whether there were ongoing new disease-specific symptoms or whether the evocative symptoms derived from other sources (e.g., having another illness or disease). Some participants stated that body signals could not always be trusted. It was sometimes hard to know whether the body signals were from the disease flare-ups or whether the internal stimuli came from another source (e.g., coming down with a cold, having lumbago, or that another disease was present).

When living a long time with an illness, you have to be especially aware of your own body. If there are any new pains, or whether you feel better, but it is more of a diagnostic process than anything else. And so you’re thinking about it: if the disease is getting worse or if there is something you can do about it. Or, you wonder if there is anything else ongoing, I mean one may even have two diseases. I had back pain in the past week, too. And then, one forgets … you forget other problems because it is so terribly painful. Ah, but it’s not a good way to get rid of such pain. (Participant 6)

Focus was to listen to the body’s signals for different bodily symptoms. For some participants, this process occurred most often during the early mornings. In the morning the body was experienced as more rigid.

I notice my body when I wake up in the morning and how it is functioning: is it as it should be, or is it worse or better or just how is it? I can only say that when I get up in the morning it feels like I’m ‘round’ under my feet. I feel very unstable when I walk, but it’s something that eventually ends. (Participant 3)

The body exhibited greatest fatigue in the evenings. Some participants said that their body felt heavy whereas others said that their body felt strained and out of balance. BA meant confronting or searching for different bodily signals (such as fatigue, stiffness, or pain). Some patients mostly focused on their pain, whereas others mostly focused on fatigue or feelings of stiffness. Focus was often on the constant evocative bodily disease-related signals and stimuli.

When I do not get reminded of it myself, so to say. And it’s then when I have pain in a joint, of course, and when I, it depends a bit on how much pain I have. I am most reminded of the pain in the morning and at night. In the morning it is a bit slow to get started, and in the evening one is simply tired from an exhausting day. (Participant 6)

## Typology II

### A reactive process on emotional triggers

Concerning this typology, the participants reported that heightened BA was triggered by different emotions. The emotions were either negatively toned (the body was not to be trusted or was seen as foreign) or positively toned (reinforcement of a positive emotion such as hope, happiness, tranquility in body signals, and curiosity). Some participants mentioned a lack of control over their condition, which was likely related to the continually unpredictable fluctuations of the disease process. The participants were also concerned about unreliable or intense disease symptoms. During disease flare-ups, some participants expressed a fear about their changing body, which led to an increased negatively toned BA. Some participants felt sadness or grief about the disease process, and some were concerned about the loss of ability caused by a diseased and degenerating body. They did not want to become a burden on their family, friends, and community. For some of the participants, the body felt no longer as a friend in that it constantly reminded them of the reduced ability to experience their self in a positive way. Other participants described having strong feelings, such as fury, anger, indignation, and rage, or felt as though they were constantly trapped inside a disabling, fatigued, and painful body. One participant explained these feelings of anger and grief in the following passage.

Yes, there is a loss. Grief. The loss of not being able to participate in activities, and grief over not having a normal functioning body. Yes, I can be pissed off. Angry at myself. But it’s the body I’m angry at most. Clumsy, or who disobeys, or do not, cannot achieve. So, when you’re sick, when you have many symptoms, one does not like one’s body. Then you have a low opinion of yourself. Then you are not as “buddies.” Then you are angry with your body. You’re angry, you hate it. (Participant 14)

This form of reactive BA sometimes involved a comparative process, i.e., a comparison with either a younger body, a healthy body, or a body as it was before the onset of the disease. This comparison triggered a considerable amount of negative emotions with the weakened body in the center of attention. Some participants experienced a longing for their former self, i.e., a body that allowed them to perform activities independently of others. They had a hard time handling the evocative negative feelings in relation to their deteriorating body.

Yes, it is primarily the hands. After all, the hands are needed for everything. For example, I cannot cook for myself anymore. These are little things that most people can manage to do … . One gets very down when one is not capable of doing even the small stuff. Nowadays, I cannot brush my hair, for example. It’s hard to get dressed. Basically, it destroys the whole day when one is unable to manage such small things. (Participant 15)

Positively toned BA meant that BA was connected to awareness of bodily strength, enjoyment, and peace or as tranquility in body signals. Some participants experienced a positively toned BA, i.e., they expressed a positive attitude to take part in physical activities or physical training.

So, now I go to a physical training center. I go once a week to a physical therapist, which I think is very good. I’ve never gone before to any physical therapy or anything throughout my sickness. But since I broke my shoulder in January, I have a really good physiotherapist. And she has taken me in a group and she has a location in the X center. There are some Italian machines, it’s called something like “X line,” or something like that. Then, I think it feels good. I can do the movements. So, it is actually very good; so, it’s positive and you feel happy when you leave. (Participant 9)

A positively toned BA was also experienced as a desire to participate in alternative treatments, which could relieve pain and discomfort.

Yes, water, one becomes enclosed. It is also a limit to the touch. Therefore, it is not always the case that one wants to be touched. When you have a lot of pain, you may not want anyone to poke at you. It may also be that you have moderate pain so you want nothing more, then that someone touches your body. I am sure that it is individually, too, where one is lying on the scale. With water, it is still a fairly straightforward form of touch. It is not as intrusive. But then there are the different touches: whether or not to take alternative treatment, so to speak. Massage and various body therapies that can calm me down. (Participant 14)

Positive BA was also experienced through living in a community with others, feelings of acceptance from others, and spending quality time with family or friends. For some participants, positive BA was linked to feelings of being cared for by others.

## Typology III

### An active process of taking an inventory of abilities

Within this typology, the participants mentioned that heightened BA was initiated by limited physical abilities, which led to the participants becoming more aware of their body. Some participants waited for “the right time” to perform whereas others were afraid to overextend themselves. Some participants attempted to postpone different activities until the future and tended to listen intently to their bodies so they could take action at the optimal time. Some daily activities were experienced as intensive and requiring a good deal of energy. At the crux of all this was their awareness of the body’s diminished energy and ability.

I think for sure that if I manage to do the right things, meet some people or do something fun outdoors. But it’s just that it gives a bit of energy. But one has to keep it up! This takes energy, too. If I would go to the cinema and watch a good movie, this requires energy as well. It becomes a catch-22 situation. (Participant 2)

Nearly every activity the participants engaged in cost a certain amount of energy. The participants tried to control their body’s abilities and energy level. The day after an episode of physical activity, the participants observed that their body felt tired and achy. To balance daily activities BA concerned listening to the body’s level of pain and fatigue signals. This was necessary so that energy could be evenly distributed throughout the day. Some participants used their energy economically, i.e., they prioritized activities. The participants experienced their faulty or weak body as a major obstacle to participating in everyday activities. It was a way to examine the body’s signals, to find out current bodily needs, and to check bodily priorities and daily status. This was an active process, aiming at determining the body’s current status and abilities.

… but you get a small case of the blues when you feel that things are not working the way you desire. One is so used to it being possible to do. I try anyway. I’m pretty stubborn, too. But that gave me more pain. Yes, I felt it, really, and still did a little too much. Afterward, I could not sleep that night because then it ached like hell. Oops! So it was like that. One has to be very careful (with activities). (Participant 12)

## Typology IV

### A shifting from BA to the outside world

In this typology the participants remarked that their attention was directed towards the world and away from BA, i.e., they were no longer mindful of their body. The participants reported that being completely absorbed in an activity in the outside world was an opportunity to be relieved from the negatively toned BA. For some participants, this shift from the body to the outside world was often initiated by the participants themselves. For other participants, it was a more passive process, i.e., they felt the shift was the result of the world intruding on their life.

I can forget about myself when I am with, for example, my grandchildren. You go into their world and play with them. If, because I can be very childish then, and play with them. And then I think why not. I’ll do it. I completely forget myself once I enter the game and get really childish. Then I can have fun and play with them. (Participant 17)

When the participants consciously distance themselves from their own body (while simultaneously directing attention to the outside world with all its meaningful activities) it helped to alleviate negatively toned BA.

I can imagine that if I go into the woods and am thinking, walking at a fast pace, and thoughts whirl around me. Then it’s not the body that’s the center of attention or important, in a way. Yeah, it doesn’t have to be thoughts either; I mean deep or intelligent thoughts. It could be something like, “oh, maybe there are more mushrooms over there?” So it’s like that type, the kind that clears your mind, the thoughts that clear your mind, maybe the body gets cleared too. (Participant 1)

Within this category, the participants reported that when they were completely absorbed by different activities they were no longer aware of the presence or existence of their body. Because disease symptoms are the focus of attention, the participants indicated that the activities were perceived as positive.

Yes, one week ago I was enjoying my life. I stayed up and watched the midnight sun. In X city, I went up to the mountains and stood there looking, fascinated by the view of the mountains, at sunset at twelve o’clock at night. So, this is something that enhances life a bit. That, despite everything, you do get there, that you’ve seen it! That one has experienced this! Then I thought afterwards that during this time I didn’t think at all about my ailments. One doesn’t think about that then. So that was a past experience that was exceptional in my mind. (Participant 3)

## Discussion

Before our study, there was little information about the meaning of BA in people with RA. Mehling et al. (2009) stressed the need for more research, a clear definition, and a comprehensive understanding of the concept of BA. Our study was conducted to gain greater insight into BA in people with RA using a qualitative phenomenological research method (EPP). We found four typologies: “A reactive process on symptoms,” “A reactive process on emotional triggers,” “An active process of taking an inventory of abilities,” and “A shifting from BA to the outside world.” We see in these typologies four possible ways of experiencing BA in persons diagnosed with RA.

The RA disease process, symptoms, and treatment may be continuous and complex (Davis, Wagner, & Groves, 2000). We found general characteristics in all 18 interviews showing that the disease had given rise to a higher degree of negatively toned BA. BA was a reactive process involving the search or control of disease-related symptoms. Some participants claimed to have examined whether the symptoms had increased or decreased, whereas others examined whether there were ongoing new disease-specific symptoms, or whether the evocative symptom resulted from having another illness or disease. Some participants believed that body signals could not always be trusted. It was sometimes hard to know whether the body signals were from disease flare-ups or whether the internal stimuli came from other sources (e.g., a common cold or some other illness). Kirmayer and Looper (2006) and Abramowitz et al. (2002) argue that BA may lead to excessive thinking about different bodily functions. Some authors further suggest that increased attention to bodily sensations (sensitivity to pain and catastrophizing) plays an important role in illness behavior in medical illness (Kirmayer & Looper, 2006). Research has also shown that BA is strongly associated with unfavorable clinical outcomes, such as the trajectory of chronic low back pain. Psychological factors (notably distress, depressive mood, and somatization) are implicated in the transition to chronic low back pain (Pincus et al., 2002). Price (1993) explored how healthy and chronically ill adults understand their body experience. Findings indicated that the resultant body paradigm is defined as the person’s explanatory model of who she or he is physically and is the result of past body experiences. The participants in our study were highly aware of their deteriorating body. Some participants compared their present condition with what they were formerly, i.e., a strong, healthy body that could perform many activities. This comparison between the present and past created a heightened negatively toned BA. Living with RA requires using different strategies to cope with a host of emotions that occur in daily life. Mental requirements and an overall need for management strategies are important (Gronning et al., 2011; Shariff et al., 2009). Tak (2006) found three major strategies of coping with daily stress: cognitive efforts (seeking peace of mind), diversional activities (getting out, doing, or looking), and assertive actions (talking and seeking). McDonald et al.’s (2012) participants reported good days and bad days, and that one must take advantage of the good days and not plan too much in advance. Our participants described a similar strategy. Some participants used cognitive efforts (e.g., seeking peace of mind) as a disclosure and living in the moment (mindfulness). Some participants used diversional activities, such as continuing to participate in life by engaging in valued and meaningful activities. Lutze and Archenholtz (2007) found that RA had a major impact on daily life and strongly influenced the individual’s mood and social life. In particular, the authors noted that feelings of helplessness and uncertainty could lead to anger and depression. The disease fluctuations with pain, fatigue, and stiffness disturbed the daily balance, causing the participants to be more aware of their body.

Results indicate that there is a case for recommendations of, and support for, health-enhancing physical activity behaviors in people with RA (Eurenius & Stenström, 2005; Stenström & Minor, 2003). In our study the participants reported that heightened BA was initiated by limited physical abilities, which led to the participants being more aware of their body. Some participants were always waiting for “the right time” to engage in activities, whereas others were afraid to overextend themselves. Some participants were trying to postpone different activities until the future, listening closely to their bodies to determine an optimal time to perform. Chronically ill individuals have profound problems with various fundamental changes in their lives. This involves experiencing the body in different ways, including dealing with disease uncertainty (Bury, 1982) and reconstructing the self (Corbin & Strauss, 1991). In our study, dealing with disease uncertainty resulted in higher BA and was considered a serious problem in daily living. McCormack (2010) posits that illness and disease can be described as a threat to the self in that illness and disease can increase the distance between what we want to manage and what we really can manage at the moment. Some participants in our study felt they could no longer express themselves in a positive manner. They wanted to think positively, but their body no longer had the energy or the physical ability to allow this functionality. RA has a major impact on daily life, especially in the way individuals with RA perform their physical activities. Melanson and Downe-Wamboldt (2003) found that physical limitations in daily life produce the largest disease-related worries.

Pain affects everyday life and may be a deterring factor in performing valued activities. It could be a struggle to determine the right balance in leisure activity because it is easy to be overactive, which might trigger an elevation in pain levels. However, the participants also viewed activities as a mediator of pain and a distraction from it (Ahlstrand et al., 2012). Because many symptoms of RA (e.g., pain and fatigue) are generally characteristic of negative emotional responses, elevated BA may be associated with poorer emotion regulation and worse perceived health. Lööf et al. (2013) found that severe fatigue in patients with RA was associated with increased BA and decreased positive affect. However, enjoyable, intellectual, stimulating, or meaningful activities can be seen as a means to alleviate negatively toned BA. Directing focus away from one’s own body by focusing instead on diverse and meaningful activities may help to facilitate positively toned BA. Thus, some evidence suggests that it should be used with caution in that distraction from, e.g., chronic pain (during a pain-inducing activity) is associated with greater post-activity pain (Goubert et al., 2004). In our study BA was a reactive process on emotional triggers. The participants in this typology posited that heightened BA was triggered by different emotions. The emotions were described as either negatively toned or positively toned. Study has demonstrated that BA may be used to monitor physical cues associated with stress and provide a start for stress reduction interventions (Baas et al., 2004). The finding in our study of a shift from the body to the outside world was initiated by either the participants themselves or by a world presenting meaningful and enjoyable activities. Studies have shown that improved BA may change a person’s attitude to his or her own body, increase the ability to listen to bodily symptoms, enhance sense of control, and provide a better understanding in how to manage chronic pain (Gard, 2005; Gustafsson et al., 2002; Gyllensten & Gard, 2008).

Still, our results indicate that in some instances it would be beneficial to turn attention away from the body and towards the outside world. This is because negatively evocative disease symptoms are often the center of attention. A comprehensive understanding of BA from the patient’s perspective could be included in future health interventions. It is important to focus on the general health of patients, which could be done by shifting attention away from body experiences to activity in the outside world (can be made conscious and even trained). By gaining knowledge about the complex concept of BA, both maladaptive and adaptive BA can be addressed with greater understanding.

### Methodological considerations

The purpose of this study was to obtain rich descriptions of lived experience of BA in persons with RA according to a phenomenological understanding of the body (Karlsson, 1993; Polit & Beck, 2012). In this study 18 interviews were conducted. Rich descriptions are not primarily based on the number of participants but are obtained by qualitative inquiry (i.e., delving more deeply and gaining unique insight) into the perceptions and experiences of the phenomenon in question (Dahlberg, Dahlberg, & Nyström, 2008; Karlsson, 1993). The majority of the participants in our study were women, which is expected because the prevalence and incidence of RA are higher in women than in men (Symmons, 2002). HL conducted all the interviews. In analyzing the interviews HL read individually all the written interview drafts and thereafter discussed the results with two of the other authors (U-BJ and JB). In the final process all the co-authors participated in the construction of the final results. This process requires a genuine openness on the part of the researchers, who do want to hear, see, and understand the phenomenon under inquiry (Karlsson, 1993). It also requires flexibility on the part of the researcher. For researchers, it is important to reflect on their own attitudes and pre-understandings of the phenomenon in question. We aimed at openness and flexibility in the research process, which was made possible through the use of several reflections, meetings, and critical discussions within the research group.

## Conclusion

In conclusion, BA can be both positively and negatively toned in patients with RA. Hence, health care workers must be able to understand when BA is positive and when it is negative from the patient’s perspective. RA resulted in a higher degree of negatively toned BA. Therefore, the ability to shift attention from BA to activity in the outside world could sometimes be beneficial for the patient’s general health.

## Scientific significance/clinical implications

Knowledge and understanding about lived experience of BA in persons with RA could be included in future interventions designed to promote patients’ general health. A phenomenological study on the experience of BA in patients with RA can complement purely medical and psychological perspectives.
